# New Neuronal Subtypes With a “Pre-Pancreatic” Signature in the Sea Urchin *Stongylocentrotus purpuratus*

**DOI:** 10.3389/fendo.2018.00650

**Published:** 2018-11-02

**Authors:** Margherita Perillo, Periklis Paganos, Teresa Mattiello, Maria Cocurullo, Paola Oliveri, Maria I. Arnone

**Affiliations:** ^1^Stazione Zoologica Anton Dohrn, Naples, Italy; ^2^Centre For Life's Origins and Evolution, University College London, London, United Kingdom

**Keywords:** Brn, islet, neurogenin, NeuroD, neuropeptide, Ptf1a

## Abstract

Neurons and pancreatic endocrine cells have a common physiology and express a similar toolkit of transcription factors during development. To explain these common features, it has been hypothesized that pancreatic cells most likely co-opted a pre-existing gene regulatory program from ancestral neurons. To test this idea, we looked for neurons with a “pre-pancreatic” program in an early-branched deuterostome, the sea urchin. Only vertebrates have a proper pancreas, however, our lab previously found that cells with a pancreatic-like signature are localized within the sea urchin embryonic gut. We also found that the pancreatic transcription factors Xlox/Pdx1 and Brn1/2/4 co-localize in a sub-population of ectodermal cells. Here, we find that the ectodermal SpLox+ SpBrn1/2/4 cells are specified as *SpSoxC* and *SpPtf1a* neuronal precursors that become the lateral ganglion and the apical organ neurons. Two of the SpLox+ SpBrn1/2/4 cells also express another pancreatic transcription factor, the LIM-homeodomain gene *islet-1*. Moreover, we find that SpLox neurons produce the neuropeptide SpANP2, and that SpLox regulates SpANP2 expression. Taken together, our data reveal that there is a subset of sea urchin larval neurons with a gene program that predated pancreatic cells. These findings suggest that pancreatic endocrine cells co-opted a regulatory signature from an ancestral neuron that was already present in an early-branched deuterostome.

## Introduction

Complex organisms have more cell types than structurally simple ones. In many cases, functionally distinct cell types show remarkably similar gene programs. This shared program can be the result of a common evolutionary ancestor cell or of convergent evolution. Another possibility is the co-option of parts of gene networks from an ancestral cell to a new cell that leads to a parallel use of the same gene repertoire. Pancreatic endocrine β-cells and neurons are an example of different cell types that share a similar gene program but exert different functions. These two cell types share many remarkable features (1–7). Some features are common to all endocrine cells, like the ability of producing polypeptide hormones (1, 2), neurotransmitters and their receptors (3, 8), while other are specific of pancreatic β-cells, like mRNA expression and chromatin methylation pattern similar to neurons (5). Many genes expressed in neuronal development are also expressed in the development of pancreatic β-cells (7), like the homeodomain protein Isl1 (9), the bHLH transcription factors neurogenins (10–12), and the homeobox transcription factor PDX1 (13, 14).

All of these aspects lead to the idea that gut cells co-opted a neuronal transcriptional program leading to the evolution of β-cells (15). While all deuterostomes have a nervous system, the discrete pancreas with fully-developed β-cells is a vertebrate specific organ (16). Therefore, gut cells must have adopted part of a neuronal program before the vertebrate ancestor appeared. When did gut cells adopt a neuronal program? Was this program common to all neurons, or rather distinctive of only some neuronal populations? An approach to answer these questions is to identify neurons that express pancreatic toolkit genes in invertebrates that have gut cells with a pancreatic-like program. Together with vertebrates, chordates include tunicates and cephalochordates. Both groups have gut cells that express insulin-like peptides (ILPs). The cephalochordate amphioxus has an ILP localized in the gut and the mesoderm (17, 18), while tunicates have cells producing ILPs in the gut and the nervous system (19–21). Besides the ILP expression pattern, there are no data on the presence of a pancreatic-like gene regulatory program in cephalochordates and tunicates.

The sister group of chordates includes the echinoderms, a clade of early-branching deuterostomes like the sea urchin *Strongylocentrotus purpuratus*. The sea urchin's simple development and available genetic tools enable comparative studies on cell specification and gene regulatory networks with other deuterostomes, such as vertebrates (22, 23).

Although sea urchin larvae do not have a true pancreas, we previously found that there are subsets of gut cells that express a pancreatic program similar to the exocrine pancreas (24). We next asked whether in the same animal there are neurons that express the transcription factors co-opted by pancreatic β-cells (summary of the genes discussed in this paper in Supplementary Table 1). For simplicity, we name the neuronal genes that are expressed in the pancreas “pre-pancreatic” genes. To date, the only cells in sea urchins known to express the neuronal transcription factors co-opted by the pancreas are a group of 3–4 ectodermal cells that express *SpLox*, the sea urchin homolog of Pdx1 (25). In vertebrate embryos, *Pdx1* is expressed in two domains: within the duodenum and the developing pancreatic endoderm (26) and in the neural cells during brain development (27). The *SpLox* ectodermal cells co-express the gene *SpBrn1/2/4* (25). *SpBrn1/2/4* is a member of the POU3 family. The human genes Brn1, Brn2, Brn4, and Oct6 are all equal co-orthologs of the sea urchin gene SpBRN1/2/4 (28). In mammals, *Brn4* is involved in the specification of glucagon producing α-cells (10, 29), while for instance Oct6 is expressed in distinct mouse brain regions (30, 31). However, besides *SpLox* and *SpBrn1/2/4* cells in the ciliary band, we lack information on the presence of neurons expressing pre-pancreatic gene. In addition, although many features of neurogenesis in sea urchins have been explained (32–36), we still know little about the diversity of neuronal subtypes. Our hypothesis is that if a pre-pancreatic neuronal subtype exists, then the gut cells that gave rise to the endocrine pancreas co-opted a gene program specific of one neuronal subtype, rather than a generic one. To this aim, we asked whether there are neurons with a gene program that resembles the pancreatic one in the sea urchin. Here we describe new subtypes of neurons that display a pre-pancreatic regulatory fingerprint and are marked by SpLox. We define these neurons as “pre-pancreatic neurons,” because these are the cells that express the gene program co-opted by the pancreas.

## Results

### SpLox is expressed in lateral ganglion and apical organ neurons

**Figure 1:**
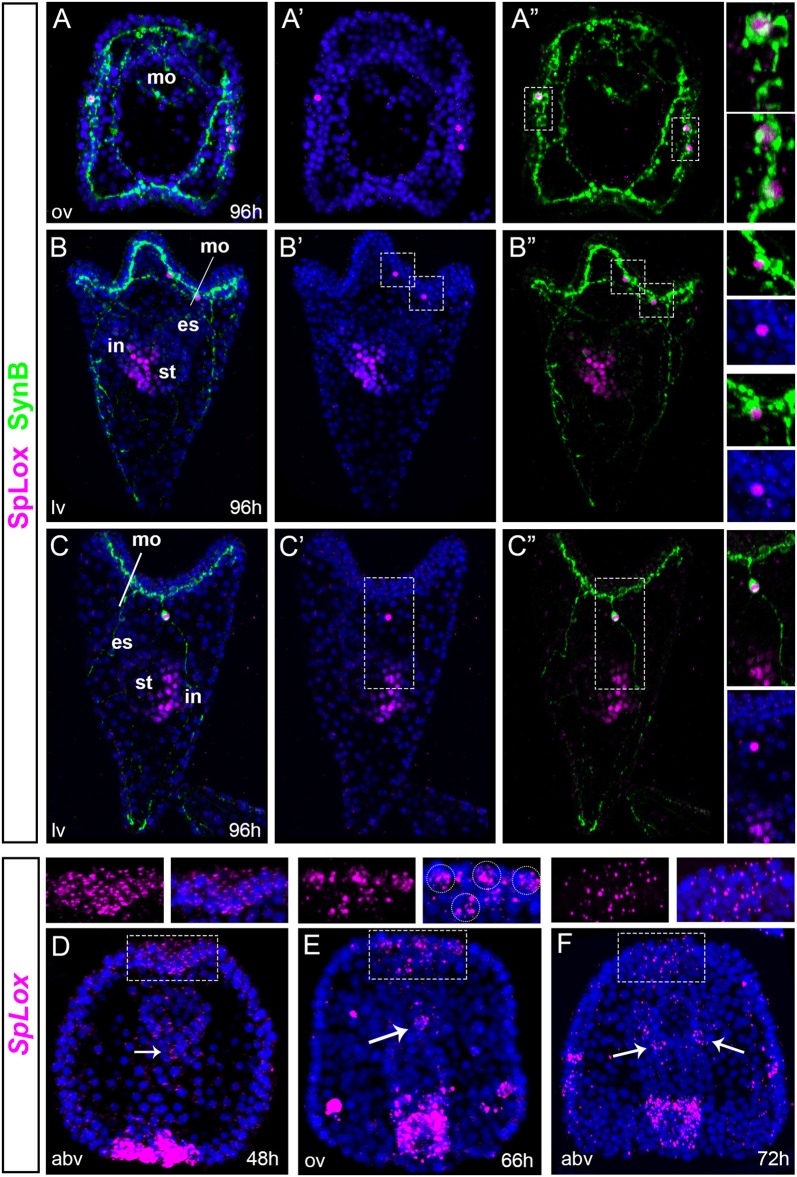
SpLox is expressed in lateral ganglion neurons. **(A–C”)** SpLox (magenta) and SynB (green) protein localization in late larvae. Nuclei are labeled blue with DAPI. In **(A”,B”,C”)** nuclei are omitted in order to see the long and interconnected network formed by the neurite projections. Insets on the right show SpLox expression in the nucleiof left lateral ganglia **(A,B)** and right lateral ganglia **(A,C)**. **(D–F)** Are fluorescent *in situ* hybridization (FISH) showing the localization of *SpLox* mRNA in the apical plate in late gastrula **(A)**, prism **(B)** and early larva **(C)** stages. White dashed line boxes are magnifications of the apical plate area. White arrowheads indicate *SpLox* localization in the foregut. All pictures are full projections of merged confocal stacks. Nuclei are stained with DAPI and depicted in blue. es, esophagus; in, intestine; mo, mouth; st, stomach; abv, aboral view; lv, lateral view; ov, oral view.

*In situ* hybridization data have previously shown that the *SpLox* gene is expressed in the endoderm (37) and also in three ectodermal cells together with *SpBrn1/2/4* (25). *SpLox* orthologs (Pdx/xlox) are involved in both nervous system and pancreas development (26, 27). Hence, *SpLox* is an important gene to investigate the regulatory state of neurons that express pre-pancreatic genes. Using an anti-SpLox antibody (38), we define the *SpLox*+*Brn1/2/4* cells in the ciliary band in relation with Synaptotagmin B (SynB), a marker of differentiated neurons in invertebrates (39). Our data revealed that the *SpLo*x+*SpBrn1/2/4*+ cells were lateral ganglion neurons (Figures 1A–C). At 96 h post-fertilization (hpf), SpLox was localized in two of the left lateral ganglia and one to two right lateral ganglion cells (Figures 1A,A',A” oral view; Figures 1B,B',B” left view; Figures 1C,C',C” right view). SpLox protein was localized in the nucleus only, consistent with its function as a transcription factor.

Next, we identified previously undescribed cells that expressed *SpLox* transcripts. First, in gastrulae we found cells within the apical plate were *SpLox* mRNA was diffused (Figure 1D). At 66 hpf the signal narrowed down to a distinct group of 3–4 cells of the apical organ (Figure 1E) and by 72 hpf *SpLox* expression was faint and diffused again (Figure 1F). Second, we identified distinct cells of the foregut that expressed *SpLox* (Figures 1E,F, white arrows). Thus, we found that the previously identified *SpLox* cells below the ciliary band are lateral ganglia neurons, and that *SpLox* is also dynamically expressed in the apical organ and foregut up to early larval stages.

### Scattered ciliary band neurons express single pre-pancreatic genes

**Figure 2:**
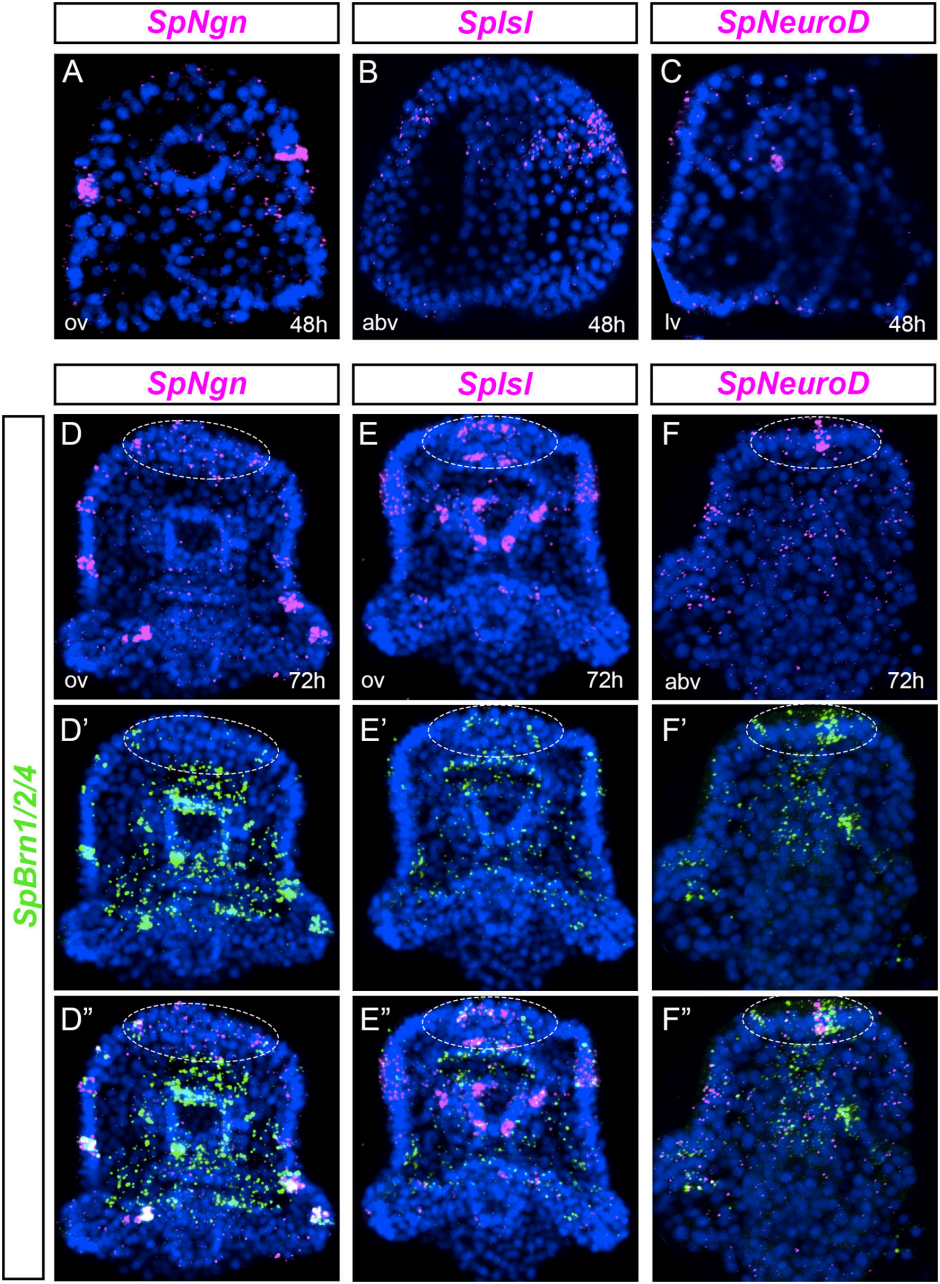
Co-expression analysis of markers of pancreatic transcription factors and *SpBrn1/2/4* define unique neurons. **(A–C)** FISH of *SpNgn, SpIsl*, and *SpNeuroD* in gastrulae. **(D–F”)** Double fluorescent *in situ* hybridization (FISH) of *SpNgn, SpIsl*, and *SpNeuroD* with *SpBrn1/2/4* combined with nuclear staining (DAPI, blue) in early larvae. White dashed-line circles highlight the apical organ region. All images are obtained as stacks of merged confocal Z sections. Split and combined channels of single confocal sections are provided to show that genes are expressed in the same cells. abv, aboral view; av, aboral view; lv, lateral view; ov, oral view.

We examined the expression of pre-pancreatic transcription factors in ectodermal cells that give rise to neurons. For some of these vertebrate genes there are multiple paralogs, while the sea urchin genome features only one paralog. Neurogenins are transcription factors expressed early in endocrine pancreas precursors (40, 41) and in neuronal differentiation (42, 43). The only sea urchin neurogenin ortholog is *SpNgn*. In gastrulae, *SpNgn* was expressed at the animal pole and in individual cells located within the ciliary band (Figure 2A). This expression increased at early pluteus stage where several *SpNgn* cells were found throughout the ciliary band (Figure 2D). *Islet* is another vertebrate gene that is expressed in the developing pancreas and in the nervous system (9, 44). In sea urchin gastrulae, *SpIsl* was expressed broadly in the oral ectoderm, particularly on the right side (Figure 2B). The expression increased during development and at the larval stage, *SpIsl* was expressed throughout the ciliary band and a few cells of the post-oral ectoderm (Figure 2E). *SpIsl* was also expressed in the foregut and in a group of cells in the upper and lower lip (Figure 2E). Last, we analyzed the expression profile of *SpNeuroD*. The SpNeuroD vertebrate orthologs are key regulators of neuronal terminal differentiation and pancreas development (45–49). In gastrulae, *SpNeuroD* was widely expressed in the oral ectoderm (Figure 2C). In early larvae, *SpNeuroD* was detected at low levels in several cells throughout the ciliary band and the apical organ (Figure 2F).

Given that *SpNgn, SpIsl* and *SpNeuroD* were all expressed in ectodermal cells, we asked if those cells were neurons that co-express *SpBrn1/2/4*, recently identified as a key gene of the sea urchin neural specification process (50). We found that most of the *SpNgn* positive ciliary band cells co-expressed *SpBrn1/2/4* (Figure 2D”). Similarly, *SpIsl* cells of the ciliary band, apical organ and foregut were *SpBrn1/2/4*+ (Figure 2E”). Last, the *SpNeuroD* apical organ cells co-expressed also *SpBrn1/2/4* (Figure 2F”). These data reveal that in early larvae there are scattered ciliary band neurons that express pre-pancreatic transcription factors, like *SpNgn, SpIsl* and *SpNeuroD*.

### A pancreatic signature is turned on in a subset of neuronal precursors

**Figure 3:**
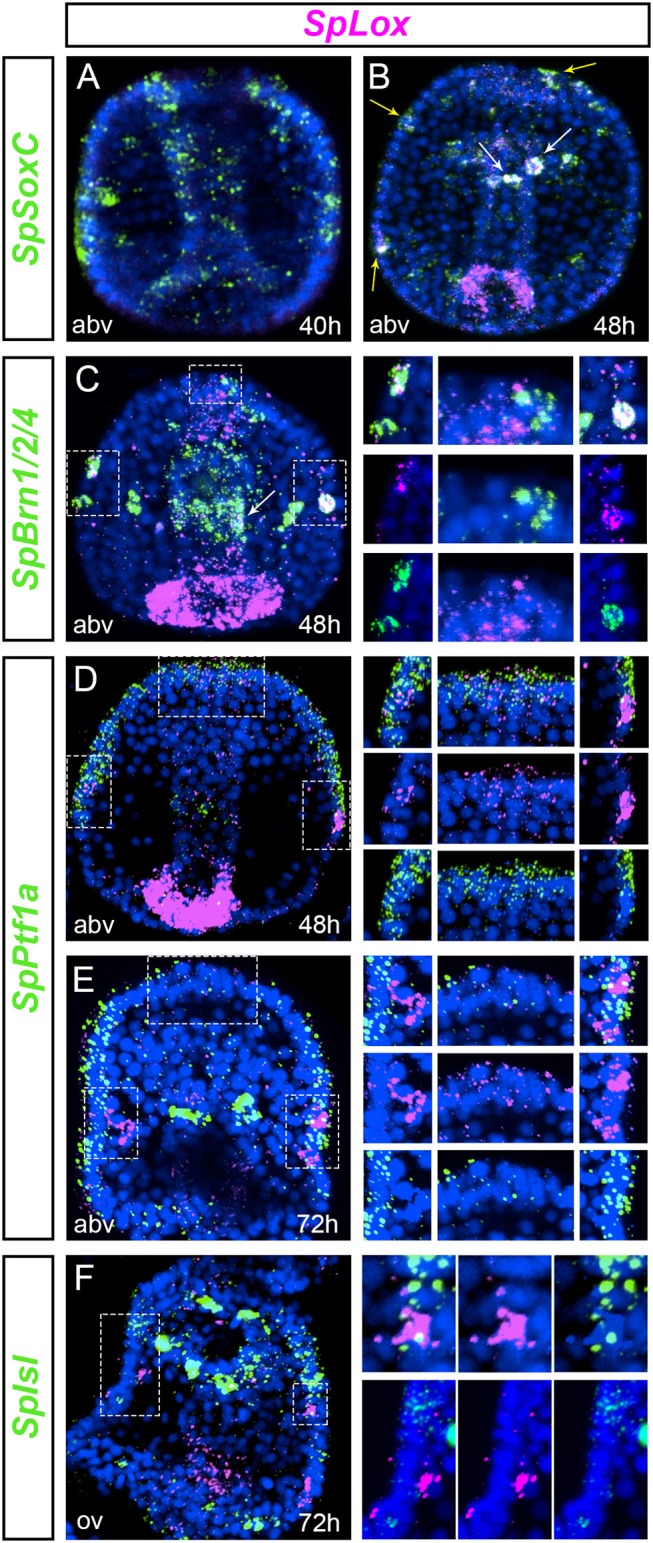
Cells with a pre-pancreatic regulatory state. **(A,B)** double FISH of *SpLox* and the proneural gene *SpSoxC* in middle to late gastrula. White arrowheads show *SpLox* co-localization with *SpSoxC* in the foregut, yellow arrowheads mark neurons in the apical plate and ciliary band that co-express *SpSoxC* and *SpLox*. **(C)** double FISH of *SpLox* and *SpBrn1/2/4* in mid gastrula. Insets on the right are magnifications of *(Continued)*

We aimed to understand whether a pre-pancreatic regulatory state was active in neuronal precursor cells. We first tested whether *SpLox* was active in progenitors that express *SpSoxC*, an early marker of neuronal development (50, 51). *SpSoxC* was already expressed in mid gastrula before SpLox is expressed in the ectoderm (Figure 3A). *SpLox* appeared in late gastrula stage in *SpSoxC* neural precursors of the apical plate and ciliary band (Figure 3B, yellow arrowheads). Additionally, the *SpLox* cells in the foregut also expressed *SpSoxC* (Figure 3B, white arrowheads). We used *SpBrn1/2/4* as a second marker for neuronal precursors. Besides the known co-expression of *SpLox* and *SpBrn1/2/4* in the ciliary band (25), we found that from gastrula stage *SpLox* and *SpBrn1/2/4* co-expressed in at least two cells of the apical plate (Figure 3C).

Next, we tested which other pancreatic genes were expressed in the *SpLox* neurons. In vertebrates, *Ptf1a* is expressed early in pancreas development in the same cells that express the *Pdx1* gene, the ortholog of *SpLox* in vertebrates (52). We previously found that ectodermal cells transiently expressed *SpPtf1a* until gastrula stage (53). We therefore tested if the *SpLox* ectodermal cells expressed also SpPtf1a. Double *in situ* hybridization revealed that cells in the apical plate and in the left and right lateral ganglion expressed both *SpLox* and *SpPtf1a* (Figures 3D,E). In particular, only in the left lateral ganglion precursors *SpPtf1a* expression was high in the adjacent cells and weak in the *SpLox* cells (Figure 3E). These same left lateral ganglion precursors also expressed SpIsl (Figure 3F). As for *SpPtf1a*, we noticed that S*pIsl* expression was higher in adjacent cells rather than in the *SpLox* neurons themselves (Figure 3F, insets on the right). Conversely, we found that *SpLox* neurons did not express SpNgn or SpNeuroD (data not shown).

Altogether, we found that apical organ, foregut and ciliary band neurons express SpLox after SpSoxC is activated. Apical plate and ciliary band cells express also SpPtf1a until gastrula stage. In the early larva at least two neurons are *SpLox*+*SpBrn1/2/4*+*SpIsl*+, likely representing a novel specialized neuroendocrine cell. Our results show that the left and right lateral ganglion neurons do not possess exactly the same molecular signature, suggesting separate functions.

### The “pre-pancreatic” neurons produce the neuropeptide SpANP2

**Figure 4:**
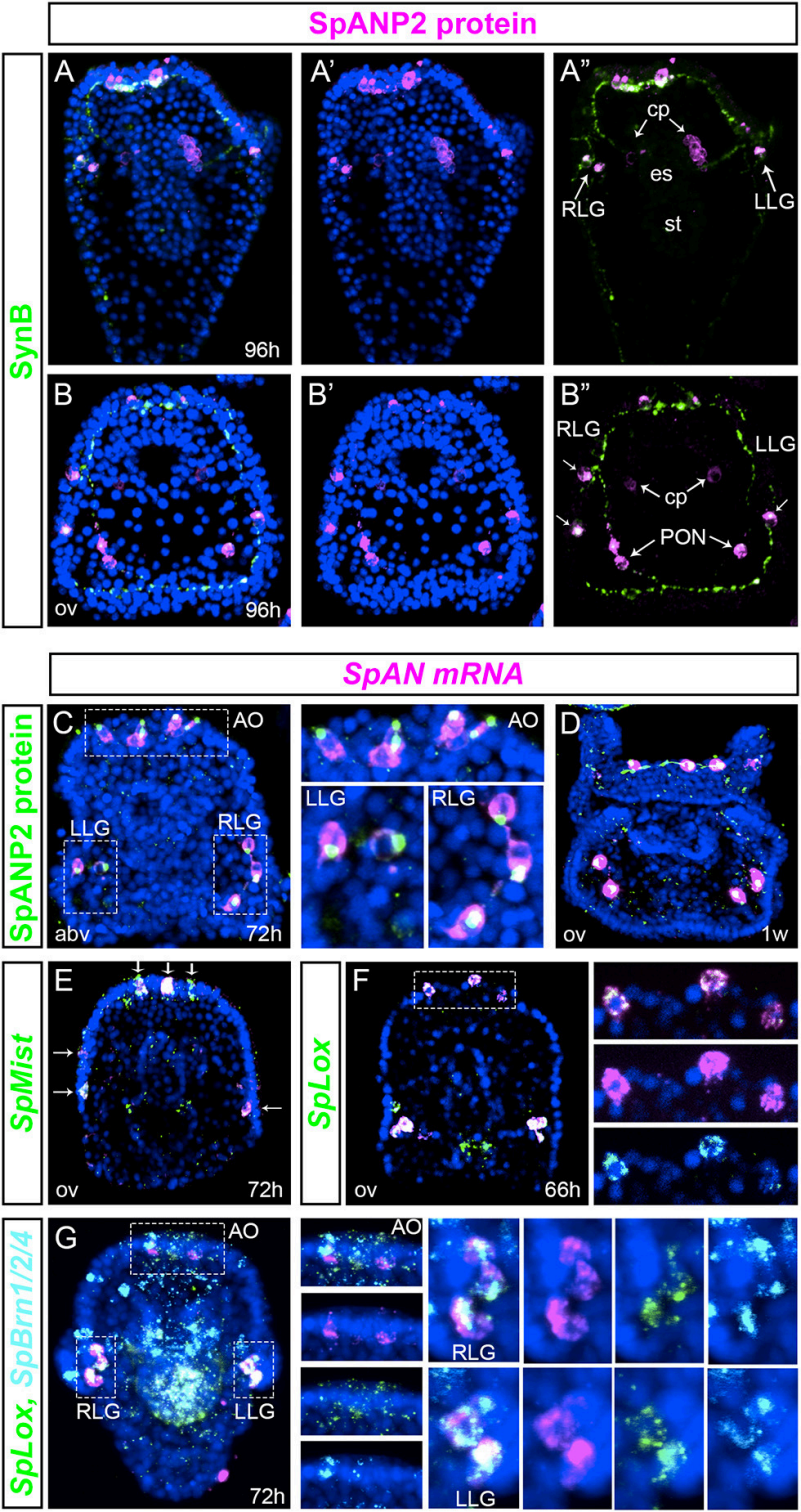
Transcript expression and protein localization of the neuropeptide SpANP2. **(A,B)”** SpANP2 (magenta) and SynB (green) immunolocalization in late larvae. **(A–A)”** is a frontal view, **(B–B)”** is a dorsal view. **(C,D)** SpANP2 transcripts (FISH, in magenta) and protein (immunofluorescence, in green) localization in early larva **(C)** and in 1-week old larva **(D)**. White dashed line boxes in **(C)** mark the apical organ region, the left and the right lateral ganglia that are magnified on the right. Note that the protein accumulates close to the neuritis extension. **(E)** Double FISH of *SpAN* (magenta) with *SpMist* (green) showing that cells producing *SpAN* mRNA are secretory endocrine cells. White arrows indicate co-expression. **(F)** Double FISH of *SpAN* (magenta) and *SpLox* (green) at prism stage (66 h). White dashed line box marks the apical organ region. Insets on the right show three distinct cells where *SpLox* and *SpAN* are co-expessed. **(G)** Triple FISH shows the expression pattern of *SpBrn1/2/4* (cyan), *SpLox* (green), and *Sppnp5* (magenta). Insets on the right show single channels for each gene. The 72 h pluteus is oriented abanal along the A/V axis. Pictures are all full projection of merged confocal stacks; nuclei are labeled blue with DAPI. AO, apical organ; cp, coelomic pouches; es, esophagus; LLG, left lateral ganglion; PON, post-oral neurons; st, stomach; RLG, right lateral ganglion; abv, aboral view); ov (oral view).

In order to identify terminal differentiation genes of the SpLox neurons, we looked at the expression of the sea urchin neuropeptides described by Woods et al. and Rowe et al. (54). In particular, *SpAN* expression pattern resembled that of *SpLox*+*SpBrn1/2/4*+ neurons. To better understand the nature of the *SpAN* expressing cells, we developed an antibody to SpANP2 protein and performed double immunostaining with anti-SynB antibody (gene name *SpAN*, protein name SpANP2). In larvae, SpANP2 localized in three to four apical organ neurons, lateral ganglia and postoral neurons (Figures 4A,B). The SpANP2+ apical organ neurons were serotoninergic and expressed also SpBrn1/2/4 (Supplementary Figure 1). Besides the ectodermal expression, SpANP2 was localized in the coelomic pouches (Figures 4A”,B”). To test whether SpANP2 immunofluorescence marked cells that produced the neuropeptide, and not target cells, we double stained larvae for *SpAN* mRNA and SpANP2 protein. We found that in early larvae the neuropeptide expression recapitulated the expression of the RNA transcripts (Figure 4C). In particular, while the mRNA was localized throughout the cells, the protein accumulated at the cell apical side (Figure 4C, white dashed line box), suggesting SpANP2 is secreted in vesicles. The same mRNA and protein expression was present also in late larvae (Figure 4D). We also found that *SpAN* neurons in the apical organ and ciliary band were secretory because they expressed *SpMist*, known marker of exocrine cells (55) (Figure 4E).

We next tested whether SpLox neurons expressed *SpAN*. We found that the SpLox apical organ and lateral ganglion neurons expressed *SpAN* (Figures 4F,G). To further test that those were the *SpLox*+*SpBrn1/2/4* neurons we performed a triple FISH experiment and confirmed that *SpAN* was expressed in all the S*pLox*+*SpBrn1/2/4* neurons (Figure 4G and insets on the right).

Taken together, our results revealed that *SpLox* marks secretory pre-pancreatic apical organ and lateral ganglion neurons that produce and secrete the neuropeptide SpANP2. Only the SpLox neurons in the foregut did not produce the SpANP2 neuropeptide.

### SpLox controls *SpAN* expression

**Figure 5:**
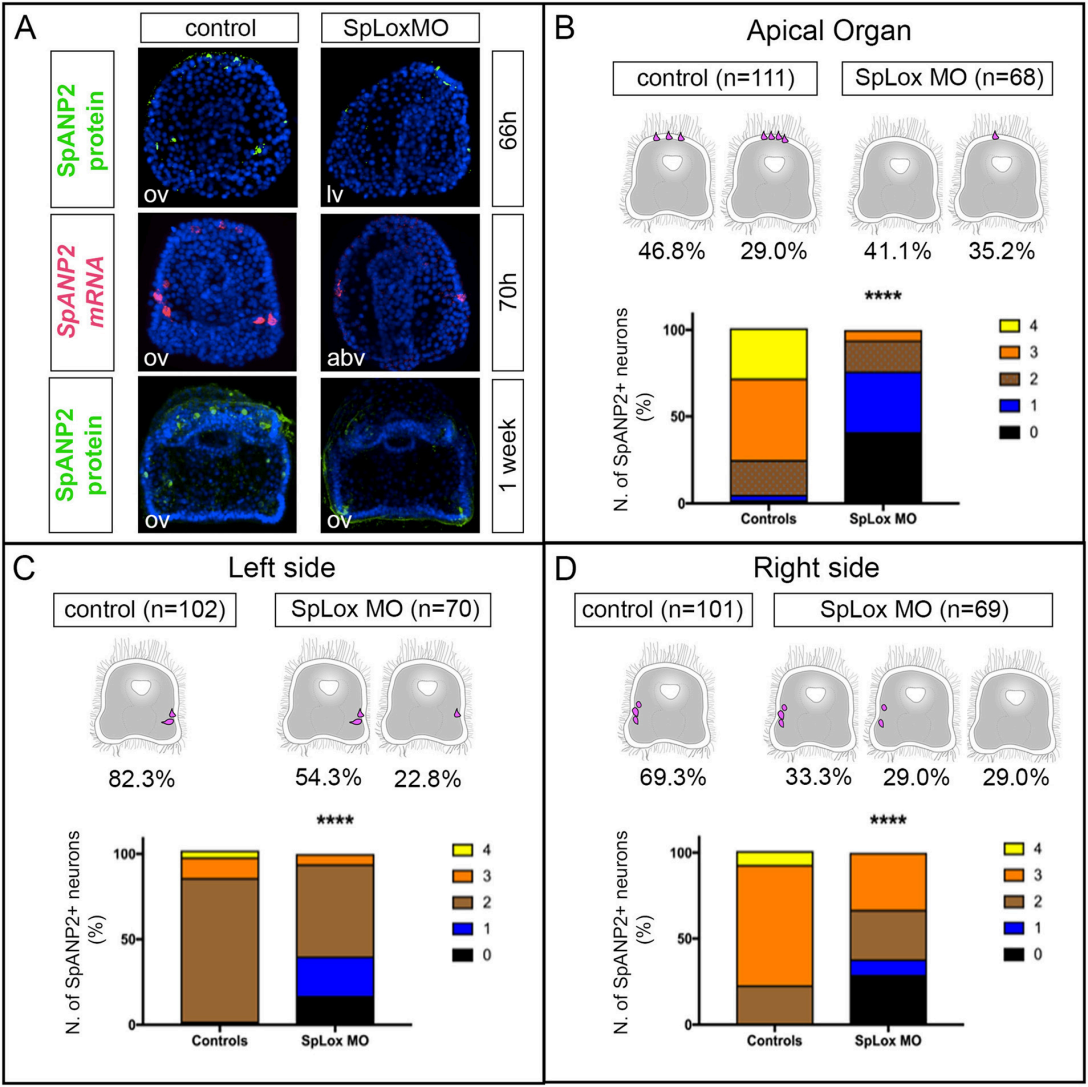
SpLox controls SpANP2 expression. **(A)** *SpAN* mRNA detected by single-color *in situ* hybridization or SpANP2 protein detected by immunofluorescence localization in controls and in larvae injected with SpLox MOs directed against the translation of *SpLox* RNA. Note that injected embryos/larvae show the typical SpLox MO phenotype of a straight gut that does not have the pyloric sphincter (38). All images are obtained as stacks of merged confocal Z sections. Nuclei are labeled blue with DAPI. **(B–D)** Quantification of the number of SpANP2 cells in the SpLox morphants shows a ^****^p < 0.0001 by Chi squared test. Cartoons of early larvae on top of the graphs summarize the most frequent phenotypes. In the graph we put together data form SpANP2+ cells at prism stage (66 h), early larvae (70 h), and 1-week old larvae. For **(C,D)** we show percentages of lateral ganglia and post-oral neurons together. abv, aboral view; lv, lateral view; ov, oral view.

Since SpLox neurons expressed also *SpAN*, we asked whether SpLox regulates *SpAN* gene expression. We used a morpholino approach to knock-down SpLox and quantified the number of neurons that expressed *SpAN* in larval stages. As previously published (37, 38), SpLox morphants are distinguished by a straight gut that lacks the pyloric sphincter. We found that in SpLox MO injected embryos there was an overall significant reduction of *SpAN* expression. For instance, control larvae had 3–4 *SpAN*+ apical organ neurons, while SpLox morphants had zero or only 1 *SpAN*+ apical organ neurons (Figures 5A,B). Similarly, SpLox morphants had fewer *SpAN*+ cells than control larvae on both the left and right sides (Figures 5A,C,D). We also observed a consistent number of larvae where there was no *SpAN* expression in at least one of the ectodermal domains (Figures 5B,C,D graphs). These results suggest that SpLox regulates *SpAN* expression in the cells where they are co-expressed: the apical organ neurons, the left and right lateral ganglia.

## Discussion

### SpLox marks a new population of neurons with a “pre-pancreatic” signature

**Figure 6:**
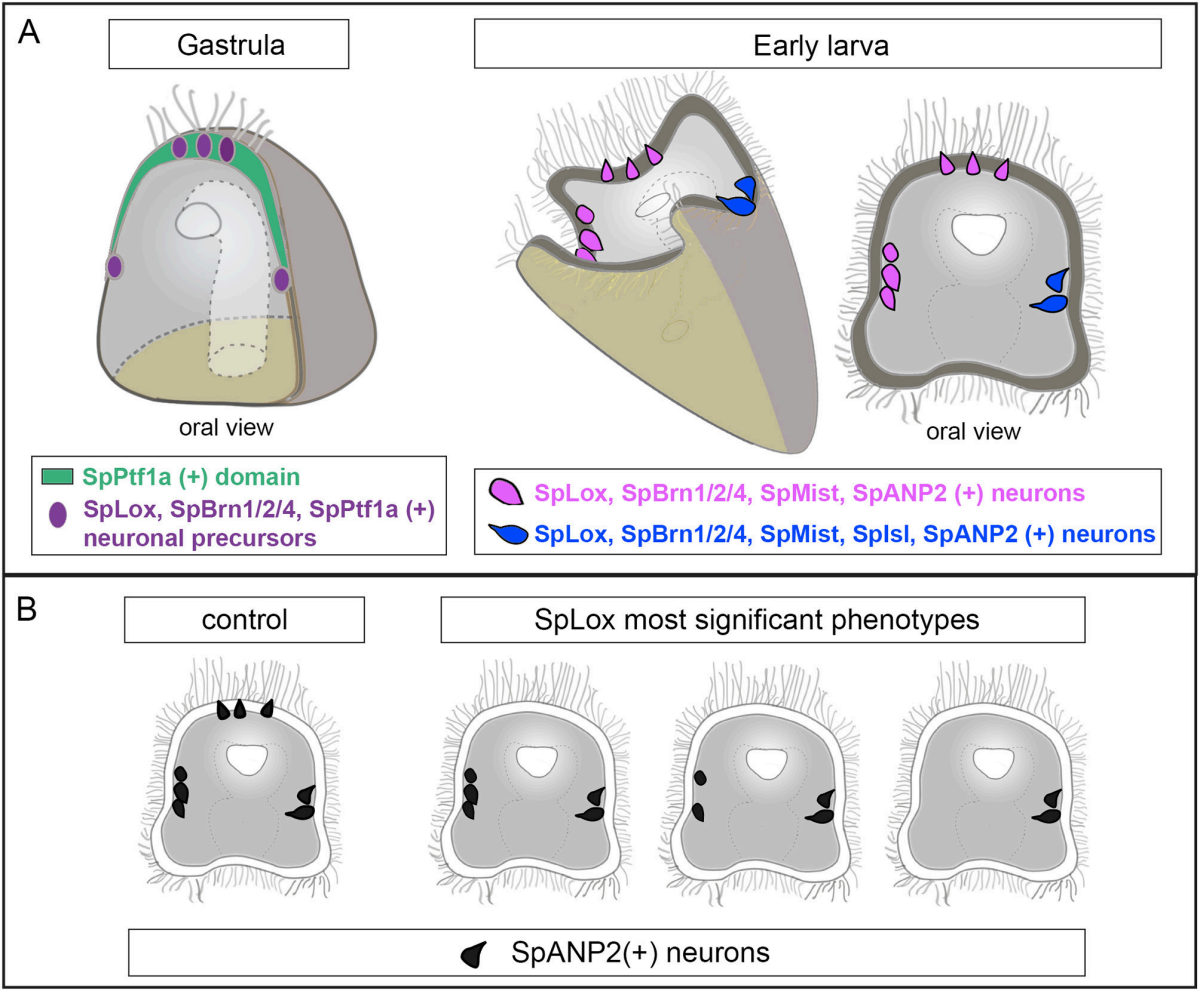
Summary of the regulatory state of the SpLox+ neurons. **(A)** Schematic representation of a sea urchin gastrula (left) and early larva (right) showing the neurons identified in this study. Neurons with the same pancreatic signature have the same color. **(B)** Cartoon showing decrease in SpANP2+ neurons in SpLox morphants. The three most frequent phenotypes are shown.

In this study we discovered a new heterogeneous population of sea urchin neurons that is marked by the ParaHox gene SpLox. It has been recently found that neuronal precursors sequentially express the transcription factors *SoxB2, SoxC*, and *Brn1/2/4* before differentiating into neurons (34, 50, 56). Our data indicate that a pre-pancreatic regulatory state marked by SpLox is active in selected neuronal precursors (summary in Figure 6A cartoon). SpLox neurons specifically express neuronal genes that the vertebrate pancreas co-opted. These “pre-pancreatic” genes are SpSoxC, SpPtf1a, and SpBrn1/2/4. First, *SpLox* is turned on in neural precursors that are *SpSoxC* positive. The vertebrate ortholog of *SpSoxC* is also expressed in the pancreatic endocrine cells during development (57). Second, *SpLox* neural precursors in the apical plate and the ciliary band transiently express *SpPtf1a*. In vertebrates, the SpPtf1a ortholog gives rise to all pancreatic progenitors (58, 59). Third, *SpLox* neurons also express *SpBrn1/2/4*, another marker of neuronal precursors (50) that is also expressed in the developing pancreas (29). The regulatory state of the *SpLox* neurons is dynamic. For instance, *SpPtf1a* might activate a pre-pancreatic program, but it is not necessary for maintaining it, while *SpBrn1/2/4* remains on until larval stages.

It is intriguing that the SpLox neurons have a similar molecular signature but a distinct spatial distribution. One hypothesis is that perhaps the apical organ neurons represent the brain component of a pancreatic circuit, while the lateral ganglia are the peripheral component. It is known that the brain is an important target of the insulin that is produced by the pancreatic endocrine β-cells (60). The sea urchin has a tyrosine kinase receptor (SpInsr) that is ortholog of the vertebrate insulin receptor (INSR) and the insulin-like growth factor 1 receptor (IGF1R). An interesting comparison is that just as INSR and IGF1R are expressed in the vertebrate brain (61), SpInsr is also expressed in the apical organ of sea urchin larvae (24). This expression pattern suggests that apical organ neurons could respond to endocrine signals from the gut as the vertebrate brain does. The most significant difference between the two *SpLox* neuronal populations is that the apical organ neurons are serotoninergic (62), while lateral ganglia are dopaminergic (63). The relationship between these two types of neurons that have a similar signature but express also different neurotransmitters merits further investigation.

### Sea urchin neurons are a heterogeneous population

SpBrn1/2/4 has been shown to be a neuronal marker (50). In this study, we found that subsets of *SpBrn1/2/4* neurons express distinct genes. For instance, *SpBrn1/2/4* ciliary band neurons express SpNgn, while the apical neurons do not always express *SpNgn*. In the sea urchin *L. variegatus, LvNgn* has been shown to have a similar expression pattern to *SpNgn* (64), but no co-expression with other transcription factors is known. *SpBrn1/2/4* neurons also express *SpNeuroD* and *SpIsl* in scattered cells. As it has been previously shown, waves of transcription factors are transiently expressed in neurogenesis (34, 50, 64). Therefore, one possibility is that cells lacking *SpBrn1/2/4* expression at a given stage might express it a few hours later. Lateral ganglia appear like symmetrical neurons localized on the left and right side of the sea urchin larva. Despite this anatomical symmetry, these neurons do not express exactly the same genes. For instance, only the SpLox left lateral ganglion neurons express also *SpIsl*. The facts that not all the lateral ganglion neurons share the same molecular signature, and that right and left lateral ganglion neurons are not symmetrical, emphasizes the complexity and diversity of neuronal types in sea urchins (summarized in Figure 6A). Altogether, our findings demonstrate that in the sea urchin larva there is a huge diversity of neuronal subtypes that has not been completely characterized.

### SpLox regulates the expression of SpANP2

We found that the novel echinoderm neuropeptide SpANP2 (Wood et al. 2018) (54) is expressed in several neurons, including the SpLox “pre-pancreatic” neurons. SpANP2 is expressed in the adult *S. purpuratus* nerve cords (65), but so far no sequence similarities with neuropeptides from other phyla have been identified (54). Its cellular localization suggests that SpANP2 is released in vesicles, in line with its role as a neurohormone. The fact that SpANP2 is expressed not only in *SpLox* neurons, but also in other cells, leads to two possible hypotheses. First, neurons with different molecular signatures could use the same mechanisms to regulate SpANP2 expression. Alternatively, different regulatory networks could control SpANP2 expression in distinct cells. In SpLox knocked down larvae, SpANP2 transcripts and protein were significantly reduced from the apical organ and in the peripheral neurons, but not all neurons were equally affected (a summary of these data is reported in Figure 6B). Therefore, our perturbation data suggest that SpLox is a general regulator of *SpANP* expression, but different gene regulatory networks could control *SpANP* expression in specific domains.

### Conclusions

Neurons and pancreatic β-cells share many remarkable features, including similar gene expression, function and physiology (1–7). It has been shown that human neural progenitors can be induced to differentiate into pancreatic cells (66), suggesting that these two cell types use a very similar gene regulatory network. Previous authors (15, 67) have discussed the idea that a multipotent pancreatic progenitor co-opted a neural genetic program. Insulin-like peptides are expressed in the nervous system and the gut of non-vertebrate chordate, like echinoderms, tunicates and cephalochordates (17, 24, 68). These findings support the idea that pancreatic cells co-opted a neural genetic program. Our results expand this idea and suggest that pancreatic cells co-opted a neuronal program from a distinct neuronal subtype, rather than a generic one. Hence, we provide additional evidence that neurons with a pancreatic signature pre-dated the appearance of the vertebrate pancreatic regulatory state. Therefore, we propose that gut cells co-opted a pre-existing pre-pancreatic program from ancestral neurons already present in a deuterostome ancestor.

## Materials and methods

### Animals

Adult *Strongylocentrotus purpuratus* were obtained from Patrick Leahy (Kerchoff Marine Laboratory, California Institute of Technology, Pasadena, CA, USA) and housed in circulating seawater aquaria at the Stazione Zoologica Anton Dohrn of Naples. Gametes were obtained by vigorous shaking of animals or by intracoelomic injection of 0.5 M KCl. Embryos were cultured at 15°C in filtered Mediterranean sea water diluted 9:1 with de-ionized water.

### RNA whole mount *in situ* hybridization

For fluorescent whole mount *in situ* hybridization (FISH), we followed the protocol outlined in (69). Triple FISH was performed as described in (70). Signal was developed with fluorophore-conjugated tyramide (1:400 reagent diluents, Perkin Elmer). Labeled probes were transcribed from linearized DNA using digoxigenin-11-UTP, fluorescein-12-UTP (Roche, Indianapolis, IN, USA), or labeled with DNP (Mirus, Madison, WI, USA) following kit instructions. *SpLox, SpBrn1/2/4, SpSoxC, SpPtf1a*, and *SpMist* probes were made as previously published [SpLox (71), SpBrn1/2/4 (25), SpSoxC (69) SpPtf1a, and SpMist (53)]. *SpIsl, SpNgn* and *SpNeuroD* probes were synthetized using the following primers: *SpIsl-F*: 5′-CGTGGACCAGACAGACTTGA-3′; *SpIsl-R*: 5′-AGTCGCTGAGTGCTTTCCAT-3′; *SpNgn-F:* 5′-TACGACAATGATGCCCAAGA-3′; *SpNgn-R*: 5′-CCGTTTCACAAAGCCATTTT-3′; *SpNeuroD-F*: 5′-CTCGCCACCTGATCTCTAC-3′; *SpNeuroD-R*: 5′-TTCCCGCCTTTCAAAATATG-3′. *SpANP*2 probe was made as published in Woods et al. 2018. Templates of all the probes were sequenced prior to probe generation and cloned in the pGEM®-T Easy Vector (Promega, Madison, WI, USA). Samples were imaged with a Zeiss 510 Meta confocal microscope.

### Immunochemistry

Larvae were fixed in 4% PFA in FSW for 15 min at room temperature, washed multiple times in phosphate-buffered saline with 0.1% Tween-20 (PBST), and incubated overnight at 4°C with either the SpLox antibody (1:500) or the SpANP2 antibody (1:250) in 1 mg/ml BSA and 4% sheep serum in PBST. To mark the nervous system, the anti-Syn antibody (1:100) was added (39). Larvae were then washed three times with PBST and incubated for 1 h at room temperature with the secondary antibodies anti-rabbit-AlexaFluor 555 (Invitrogen) or anti-mouse 488 (Invitrogen) diluted 1/100 in 1 mg/ml BSA in PBST. After being washed in PBST, larvae were mounted for imaging with a confocal microscope (Zeiss 510Meta).

### Perturbation experiments with MO injection

Translation-blocking antisense MO against SpLox was used at a concentration of 2 mM as published in (37) and (38). For each experiment 300 eggs were injected with ~2–4 pl of oligonucleotide injection solution. Each experiment was repeated at least three times. Note that the SpLox morpholino exhibits an unique phenotype that lacks the pyloric sphincter, as it has been previously shown (38). As a negative control, fertilized eggs were injected with the standard control morpholino (GeneTools, Pilomath, OR) and compared side-by-side with knockdown embryos.

## Author contributions

MP conception and design, acquisition of data, analysis and interpretation of data, drafting and revising the article, PP acquisition of data; analysis and interpretation of data, TM and MC: acquisition of data, PO analysis and interpretation of data, revising the article, contributed unpublished essential reagents, MA conception and design, analysis and interpretation of data, drafting or revising the article.

### Conflict of interest statement

The authors declare that the research was conducted in the absence of any commercial or financial relationships that could be construed as a potential conflict of interest.

## Supplementary material

Supplementary Figure 1 SpANP2 is localized in serotoninergic apical organ neurons. Serotonin, Synaptotagmin B and SpANP2 immunofluorescence. The picture is a full projection of merged confocal stacks. Nuclei are stained with DAPI and depicted in blue.

Supplementary Table 1 Summary of the genes discussed in the present paper.
