# Supplementary figures and images for: New Neuronal Subtypes With a “Pre-Pancreatic” Signature in the Sea Urchin Stongylocentrotus purpuratus
Source: Front Endocrinol (Lausanne). 2018 Nov 2;9:650. doi: 10.3389/fendo.2018.00650 (PMC6224346; doi:10.3389/fendo.2018.00650)

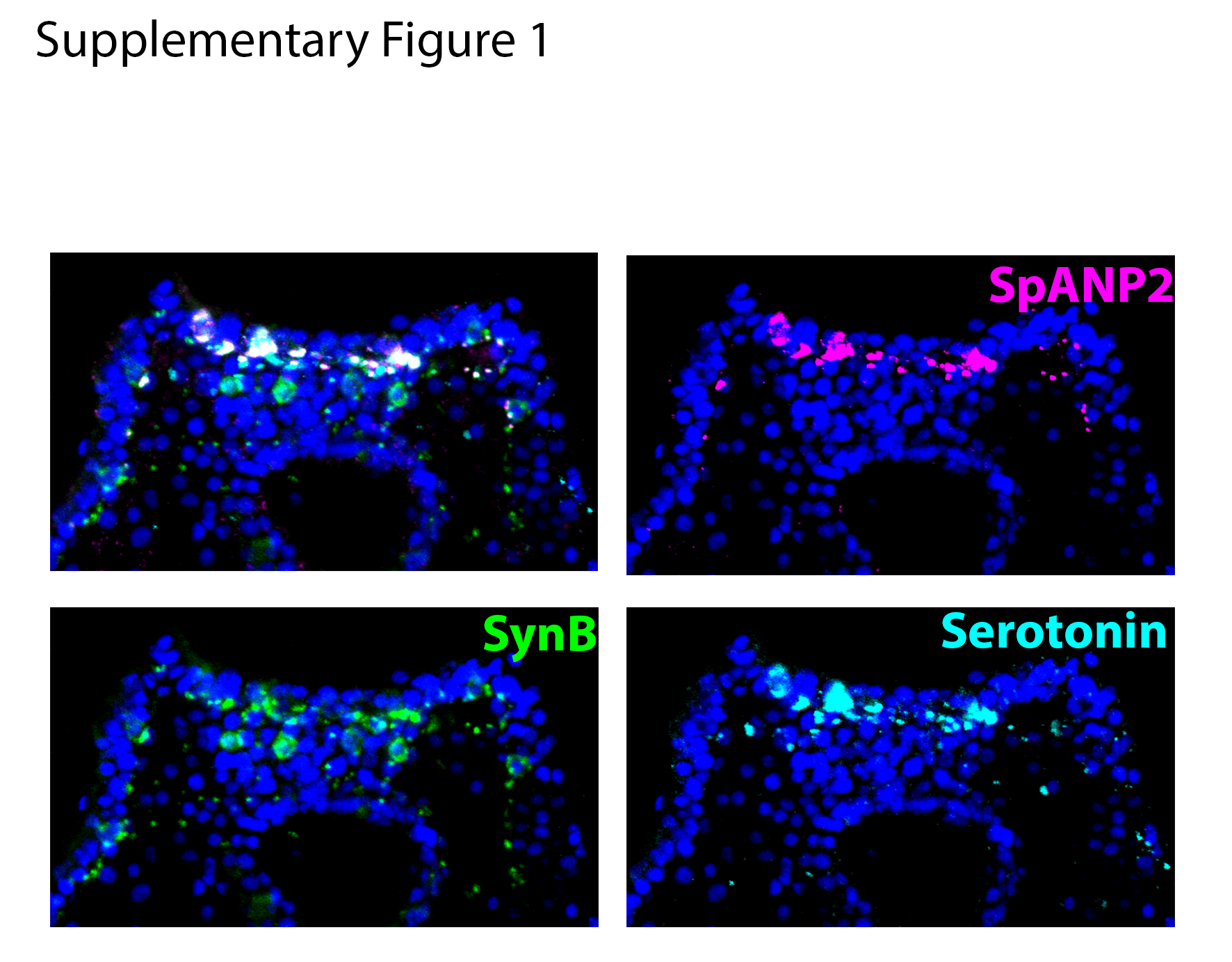

Supplement: Supplementary Figure 1 — SpANP2 is localized in serotoninergic apical organ neurons. Serotonin, Synaptotagmin B and SpANP2 immunofluorescence. The picture is a full projection of merged confocal stacks. Nuclei are stained with DAPI and depicted in blue. [file Image_1.TIF]
