# Supplementary material for: New Neuronal Subtypes With a “Pre-Pancreatic” Signature in the Sea Urchin Stongylocentrotus purpuratus
Source: Front Endocrinol (Lausanne). 2018 Nov 2;9:650. doi: 10.3389/fendo.2018.00650 (PMC6224346; doi:10.3389/fendo.2018.00650)
Supplement: Supplementary Table 1 — Summary of the genes discussed in the present paper. [file Table_1.DOCX]

**Supplementary Table 1**

| **Gene name** | **Vertebrate orthologs** | **Description** | **Domain of expression in the vertebrate pancreas** | **Domain of expression in the vertebrate nervous system** | **Domain of expression in sea urchin** | **References** |
| --- | --- | --- | --- | --- | --- | --- |
|  |  |  |  |  |  |  |
| SpBrn1/2/4 | Brn1,Brn2, Brn4, Oct6 | Brain-specific homeobox/POU3 domain family | Pancreas α-cells | Developing brain | Foregut; ciliary band and apical organ neurons | (13,19,25,44,67-69) |
| SpIsl | Isl1 | Islet1 (LIM-homeodomain) | Pancreatic dorsal bud, pancreas endocrine cells | Motoneurons, periphereal neurons, subsets of brain neurons | Neurons of the ciliary band, upper lip, foregut, anus, stomach | (34,70,71) |
|  |  |  |  |  |  |  |
| SpLox | Pdx1 (Ipf1, IDX1,xlox) | Pancreatic and duodenum homeobox 1 (Parahox homeodomain) | Early pancreatic progenitors; pancreas endocrine cells | Central Nervous System | Hindgut, piloric sphincter, apical organ , lateral ganglia, foregut | (21,32,46) |
|  |  |  |  |  |  |  |
| SpMist1 | Mist1 | Muscles, intestine and stomach expression 1 (bHLH) | Pancreas exocrine cells | n.a. | Acinar-like cells, apical organ, selected neurons | (47,72) |
|  |  |  |  |  |  |  |
| SpNeuroD | NeuroD1/Beta2 | Neuronal differentiation 1 (bHLH) | Pancreas endocrine cells | Developing cerebellum and hippocampus, adult brain | Oral ectoderm, upper lip and forefut neurons, stomach | (39,41) |
|  |  |  |  |  |  |  |
| SpNgn | Ngn3 | Neurogenin 3 (bHLH) | Pancreas endocrine progenitor cells | Cranial sensory neurons | Apical organ and ciliary band neurons | (35,37,58,73-75) |
|  |  |  |  |  |  |  |
| SpPtf1a | PTF1/p48 | Pancreatic transcriptional factor1 (bHLH) | early pancreatic progenitors; pancreas exocrine cells | Developing nervous sytem, GABAergic neurons in the cerebellum | Ectoderm and upper stomach | (47,76,77) |
|  |  |  |  |  |  |  |
| SpSoxC | Sox4 | SRY (sex determining region Y)-box 4 (High mobility group) | Pancreas endocrine cells and a subset of exocrine cells | Central Nervous System | Neurons, coelomic pouches, anal and pyloric sphincter | (28,44,51,78) |
|  |  |  |  |  |  |  |
